# Insulin B-chain hybrid peptides are agonists for T cells reactive to insulin B:9-23 in autoimmune diabetes

**DOI:** 10.3389/fimmu.2022.926650

**Published:** 2022-08-10

**Authors:** Janet M. Wenzlau, James E. DiLisio, Gene Barbour, Mylinh Dang, Anita C. Hohenstein, Maki Nakayama, Thomas Delong, Rocky L. Baker, Kathryn Haskins

**Affiliations:** ^1^ Department of Immunology and Microbiology, School of Medicine, University of Colorado, Aurora, CO, United States; ^2^ Department of Pharmaceutical Sciences, Skaggs School of Pharmacy, University of Colorado, Aurora, CO, United States; ^3^ Department of Pediatrics-Barbara Davis Center, School of Medicine, University of Colorado, Aurora, CO, United States

**Keywords:** type 1 diabetes, autoimmune diabetes, T cell, insulin, B:9-23, NOD mouse, HIPS

## Abstract

Insulin is considered to be a key antigenic target of T cells in Type 1 Diabetes (T1D) and autoimmune diabetes in the NOD mouse with particular focus on the B-chain amino acid sequence B:9-23 as the primary epitope. Our lab previously discovered that hybrid insulin peptides (HIPs), comprised of insulin C-peptide fragments fused to other β-cell granule peptides, are ligands for several pathogenic CD4 T cell clones derived from NOD mice and for autoreactive CD4 T cells from T1D patients. A subset of CD4 T cell clones from our panel react to insulin and B:9-23 but only at high concentrations of antigen. We hypothesized that HIPs might also be formed from insulin B-chain sequences covalently bound to other endogenously cleaved ß-cell proteins. We report here on the identification of a B-chain HIP, termed the 6.3HIP, containing a fragment of B:9-23 joined to an endogenously processed peptide of ProSAAS, as a strong neo-epitope for the insulin-reactive CD4 T cell clone BDC-6.3. Using an I-A^g7^ tetramer loaded with the 6.3HIP, we demonstrate that T cells reactive to this B-chain HIP can be readily detected in NOD mouse islet infiltrates. This work suggests that some portion of autoreactive T cells stimulated by insulin B:9-23 may be responding to B-chain HIPs as peptide ligands.

## Introduction

Insulin has long been considered to be a predominant and disease-initiating antigen in Type 1 Diabetes (T1D) in humans and in the non-obese diabetic (NOD) mouse model of autoimmune diabetes (1). There is evidence for insulin as a primary target antigen in T1D and the NOD mouse with respect to both humoral and T cell mediated autoimmunity. NOD mice and patients with T1D exhibit insulin autoantibodies early in the disease process (2–7). Children displaying high titers of insulin autoantibodies at a young age, especially in combination with other types of ß-cell autoantibodies, typically experience a rapid progression to disease onset (8). Insulin-reactive T cells are a major component of islet infiltrates of pre-diabetic NOD mice (9–11) and can be detected as early as six weeks of age (12, 13). The pathogenicity of insulin-reactive CD4 T cells has been demonstrated *via* adoptive transfer of T cell clones isolated from the islets of prediabetic NOD mice into NOD and/or NOD.*scid* mice (9, 14, 15). T cells reactive to proinsulin have likewise been identified and cloned from the peripheral blood of T1D patients (16–22), and from the residual islets of deceased organ donors with T1D (23–26). The specificity of human insulin-reactive T cell clones includes native and altered proinsulin epitopes within the insulin B-chain, C-peptide and A-chain (16, 17, 23–25, 27–30).

Various epitopes have been mapped within proinsulin in both T1D and the NOD mouse (24, 31), but the B:9-23 sequence from the insulin B-chain has been the most widely studied. This sequence was found to be an epitope for several NOD islet-derived T cell clones (32) and has subsequently been regarded as the principal antigenic region of insulin (33, 34). The native B:9-23 sequence is required for disease initiation in the NOD mouse as mutation of one amino acid (B16Y>A), a key residue for the MHC-II I-A^g7^/T cell receptor interaction, protects NOD mice from diabetes, ablates islet infiltration, and prevents development of insulin autoantibodies (34). Unanue and colleagues have characterized two distinct types of insulin-reactive CD4 T cells with divergent functions, targeting the B:12-20 or B:13-21 epitopes within B:9-23 (30). The importance of the insulin B:9-23 epitope has also been demonstrated by responses of CD8 T cells specific for insulin B:15-23, the ligand for the diabetogenic CD8 T cell clone G9C8, in work by Wong et al., that provided the first example of an antigen in autoimmune diabetes targeted by both CD4 and CD8 T cells (35–37).

Identifying the antigenic ligands for autoreactive CD4 T cells in autoimmune diabetes has been a major focus of our studies. Among our panel of diabetogenic CD4 T cell clones, two prototypic examples, BDC-2.5 and BDC-6.9, were derived from diabetic NOD mice and selected through growth cycles with islet cells as antigen. We recently discovered that the cognate ligands for these two clones are unique post-translational modifications in the form of hybrid insulin peptides (HIPs), non-genomically encoded peptide sequences comprised of a C-peptide fragment at the N-terminus fused to sequences from endogenous proteolytically processed β-cell secretory granule proteins (28, 38, 39). The 2.5HIP consists of an insulin C-peptide fragment covalently bound to the WE14 peptide, a cleavage product from chromogranin A (ChgA), whereas the 6.9HIP has the same C-peptide sequence bound to a proteolytically processed region of pro-islet amyloid polypeptide (IAPP) (40). These HIPs and others have been identified in mouse and/or human islets (41). In addition, HIP-reactive T cells have been characterized among PBMC from T1D patients (38, 42, 43) as well as in the islets of deceased T1D organ donors (25, 28, 38).

**Figure 1:**
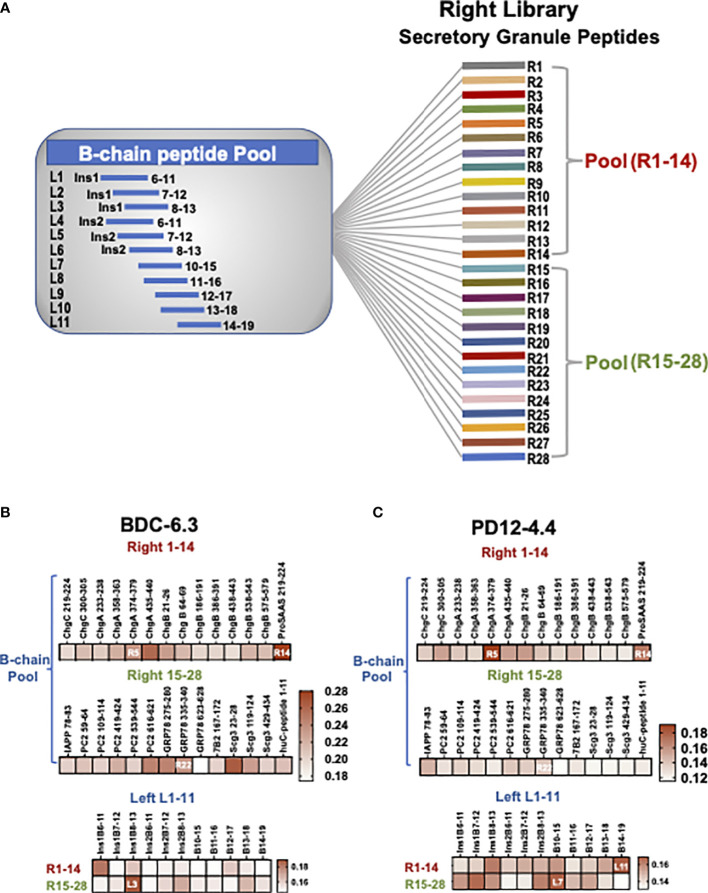
B-chain HIP crosslinking library scheme and IFN-γ ELISAs for T cell clone BDC-6.3 and PD12-4.4. **(A)** In the Left Library, 11 individual B-chain peptides form peptide bonds with two pools of right secretory granule peptides (pools R1-14, R15-28). The Right Library is comprised of 28 individual right peptides that form peptide bonds with the pool of 11 B-chain left peptides. **(B, C)** Heat maps for IFN-γ ELISA screening of T cell clones BDC-6.3 and PD12-4.4 with B chain Right and Left HIP libraries. Data is shown as absorption @ 415 nm minus background for a single screen for each T cell clone.

A subset of T cell clones in the BDC panel, and a T cell clone PD12-4.4 described by Wegmann and colleagues (32), react to native insulin and/or to insulin B-chain and B:9-23. Because responses by these clones to insulin and the B:9-23 sequence are variable and orders of magnitude lower when compared to responses to HIPs from T cell clones like BDC-2.5, we hypothesized that the peptide ligands for the insulin-reactive clones were also HIPs containing sequences from B:9-23 (instead of insulin C-peptide) joined to other granule protein sequences. In the current study we screened combinatorial B-chain HIP libraries (**Figure 1**) using a small panel of insulin-reactive T cell clones to investigate the role of B-chain HIPs as new epitopes in autoimmune diabetes. We have identified a potent B-chain HIP antigen which when loaded on tetramers detects a distinct population of T cells within NOD islets.

## Materials and methods

### Mice

NOD (NOD/ShiLtJ (# 001976)) and NOD.*scid* (NOD.Cg-Prkdc*scid*/J (# 001303)) mice were obtained from The Jackson Laboratory. The NOD/Haskins colony was derived from three strains in 1995: the original NOD/ShiJcl (Central Laboratory for Experimental Animals Japan, Inc) developed in 1980, the NOD/ShiLtJ (The Jackson Laboratory (#001976)) and the NOD/MrkTac (Taconic) strains. The NOD/Haskins strain has been interbred for 67 generations. Mice were monitored for development of diabetes by urine glucose testing (Diastix: Bayer) and validated by blood glucose testing with the OneTouch Ultra glucometer (LifeScan). Mice were defined as diabetic when blood glucose levels exceeded 15 mmol/L (270 mg/dL) on two consecutive measures. Mice were bred and housed in a pathogen-free environment at the University of Colorado School of Medicine and all experiments were conducted according to guidelines approved by the Institutional Animal Care and Use Committee.

### Isolation and culture of T cell clones

T-cell clone BDC-6.3 was originally isolated from NOD mouse islets and restimulated every 2 weeks as previously described (44) with porcine insulin (Sigma-Aldrich) and IL-2 (2.5%) EL-4 cell supernatant. T cell clone PD12-4.4 is an insulin-reactive T cell clone originally produced in the lab of Wegmann and colleagues (9).

### T cell receptor sequencing

Total RNA was extracted from T cell clones with a RNAeasy Mini Kit (Qiagen) and cDNA synthesized using a SMARTer RACE cDNA Amplification Kit (Clontech). Full length TCR α and β chain variable region genes were amplified using nested PCR followed by sequencing on the NovaSEQ 6000 sequencer as previously reported (12, 45). TCR sequencing data is listed in **Table S1**.

### Antigen assays

T cell clones (2 x 10^4^) were co-cultured with peritoneal exudate cells (2.5 x 10^4^) as antigen presenting cells and synthetic peptides (CHI Scientific, Genescript) or whole porcine insulin (Sigma-Aldrich) for 48 hrs in 0.25 ml in 96-well plates. Interferon gamma (IFN-γ) secretion in the culture supernatant was measured *via* ELISA using anti-mouse IFN-γ (BD Pharmingen) to coat ELISA plates, biotin-labeled anti-mouse IFN-γ (BD Pharmingen), streptavidin-peroxidase (Sigma), and 2,2’-Azino-bis(3-ethylbenzothiazolin-6-sulfonic acid) diammonium salt for colorometric detection. Absorption was quantified at 415 nm on a microplate reader (iMARK, BIORAD) (46, 47).

### Adoptive transfer of CD4 T cell clones

To confirm diabetogenicity, T cell clones were expanded in secondary cultures with IL-2 four days post-restimulation and 1 x 10^7^ cells were injected intraperitoneally into 6-18 day-old NOD, NOD.*scid* or NOD/Haskins recipient mice which were monitored for disease incidence.

### Peptides and B-chain HIP libraries

Peptides were obtained at a purity >95% (CHI Scientific or GenScript) and are listed in **Figure S1**. The Left and Right Libraries are composed of 12 amino acid peptide pools: each HIP is comprised of 6 amino acids from insulin B-chain (left) and 6 amino acids from different secretory granule proteins (right). As shown in the left panel of **Figure S1**, all B-chain left peptides were acquired with acetyl-blocked N-termini, three arginine (R) residues for solubility, and a glycine (G) residue as a spacer. The right peptides are derived from proinsulin, islet amyloid polypeptide (IAPP), chromogranins A (ChgA) B (ChgB), and C (ChgC), secretogranin 3 (Scg3), prohormone convertase 2 (PC2), neuroendocrine protein 7B2, ProSAAS, glucose regulated protein 78 (GRP78), and neuropeptide Y (NPY1). To generate the left library, eleven individual B-chain left peptides were activated and cross-linked to two pools (14 peptides/each) of right peptides, shown schematically in **Figure 1A** as described previously (28). Reciprocally, to generate the right library, twenty-eight individual right peptides were cross-linked to a pool of eleven activated B-chain peptides. Subsequent screens were conducted with synthetic B-chain HIPs consisting of 14 amino acids to include adjacent amino acids that could enhance antigen binding.

### Flow cytometry

Antibodies used in flow cytometry surface marker phenotyping experiments were: anti-CD45 BUV395 (clone 30-F11) (BD Biosciences), anti-CD4 BV711, BV786 (clone GK1.5) (BD Biosciences) or FITC (clone RM4-5) (BioLegend), anti-Ly6G BB700 (clone 1A8) (BD Biosciences), anti-CD11c BB700 (clone HL3) (BD Biosciences), anti-CD11b BB700 (clone M1/70) (BD Biosciences), anti-CD19 BB700 (clone 1D3) (BD Biosciences), anti-CD8 BB700 (clone 53-6.7) (BD Biosciences), anti-CD44 BV711 (clone IM7) (BioLegend), and anti-CD62L FITC (clone MEL-14) (BioLegend). The fixable viability dye eFluor780 (eBioscience) was used for live/dead discrimination. Gating strategy for lymphocytes was determined by gating singlets using FSC-H by FSC-A and lymphocytes were gated using FSC-A by SSC-A. Live CD4+ cells assessed for tetramer staining were CD45+, lineage- (Ly6G, CD11c, CD11b, CD19, CD8). An example of gating strategy can be found in the **Figure S2**. Samples were analyzed on a Cytek Aurora flow cytometer (5 lasers) and data was analyzed using FlowJo software V10. Tetramers labeled with either PE or APC were acquired from the NIH Tetramer Core and were used to stain T cells at 37°C for 1 hr as reported previously (46). I-A^g7^ tetramers used in this study contained the following peptides: HEL_11-25_, 2.5HIP (28), two insulin B:9-23 mimotope peptides (insp8G and insp8E) (11) and 6.3HIP_1-11_ (B_13-19_/ProSAAS_219-222_).

## Results

### Diabetogenic T cell clone responses to insulin and the B chain B:9-23 epitope require high concentrations of antigen

The NOD-derived BDC T cell clones, originally isolated from co-culture of primary NOD spleen and lymph node cells (44), are maintained in culture with islet cells or extracts from β-cell tumors, which contain only very small amounts of antigen. Most of these T cell clones exhibit high sensitivity and specificity to islet peptide antigens, but *in vitro* responses of the insulin-reactive BDC CD4 T cell clones to the B:9-23 peptide, as well as whole insulin, are variable and low level (e.g., near background). Expansion of the insulin-reactive T cell clones requires stimulation with high concentrations of insulin, and in antigen assays (**Figure S3**) concentrations of whole insulin (10 mM and 1.0 mM) and B:9-23 peptide (10 and 1.0 µM) were required to observe responses from the insulin-reactive T cell clones PD12-4.4 and BDC-6.3. Disease relevance of the insulin-reactive T cell clones was demonstrated by the capacity to efficiently transfer diabetes into NOD or NOD.*scid* neonatal (< 3 wks of age) recipient mice (**Table 1**).

**Table 1:** Features of insulin-responsive T cell clones.

Clone	TCR α	TCR ß	Insulin/B:9-23	DT NOD	DT NOD.*scid*	DT NOD/Haskins	CDR3 α	CDR3 ß
BDC-6.3	TRAV5D-4	TRAB5	**+**	N/A	N/A	5/7 13-23 days	CAASAVGSGGSNYKLTF	CASSQEGGGNEQYF
PD12-4.4	TRAV5D-4	TRAB5	**+**	7/9 9-24 days	½ 16 days	4/6 9-24 days	CAASASGGSNYKLTF	CASSQDTNTGQLYF

Listed are TCR Vα, Vβ and CDR3 α and β designations and T cell stimulation scores (+ or -) for responses to insulin and B-chain peptide B:9-23 for the set of two T cell clones. Diabetogenicity of each clone was assessed by injecting 6-18 day-old indicated recipient mice with 1 X 10^7^ T cells and monitoring for incidence of diabetes 3-4 wks post transfer. Disease transfer (DT) numbers represent the number of diabetic mice/total number of mice injected.

### B:9-23-responsive T cell clones are stimulated by B-chain HIP antigen pools

To test our hypothesis that the peptide ligands for insulin-reactive T cell clones could consist of a portion of B:9-23 fused to other secretory granule protein cleavage products, combinatorial B-chain HIP libraries were assembled representing 308 potential HIPs. Left and right peptide library pools were generated to test novel B-chain HIP candidate epitopes. The “Left Peptide Library” contains individual B-chains (11 peptides) on the amino termini of the HIPs cross-linked to two pools of abundant secretory granule peptides (14 peptides each) on the carboxy (C-) termini of the HIPs (41) to yield 28 pools. The “Right Peptide Library” contains individual secretory granule peptides (28 peptides) on the carboxy (C-) termini of the HIPs cross-linked to a pool of the eleven B-chain peptides on the amino termini to yield 28 pools (shown schematically in **Figure 1A** and detailed in Materials and Methods). Criteria for B-chain (“left”) peptides included a leucine (L), isoleucine (I) or valine (V) at either of the last two amino acid residues at the HIP junction as these amino acids are predicted to have high affinity for pocket 4 (p4) of the I-A^g7^ binding groove. All C-terminal “right” peptides (**Figure S1**) contained either an aspartic acid (D) or glutamic acid (E) residue located four or five amino acids from the N-terminus as negatively charged amino acids most often occupy pocket 9 (p9) of I-A^g7^ (48).

**Figure 2:**
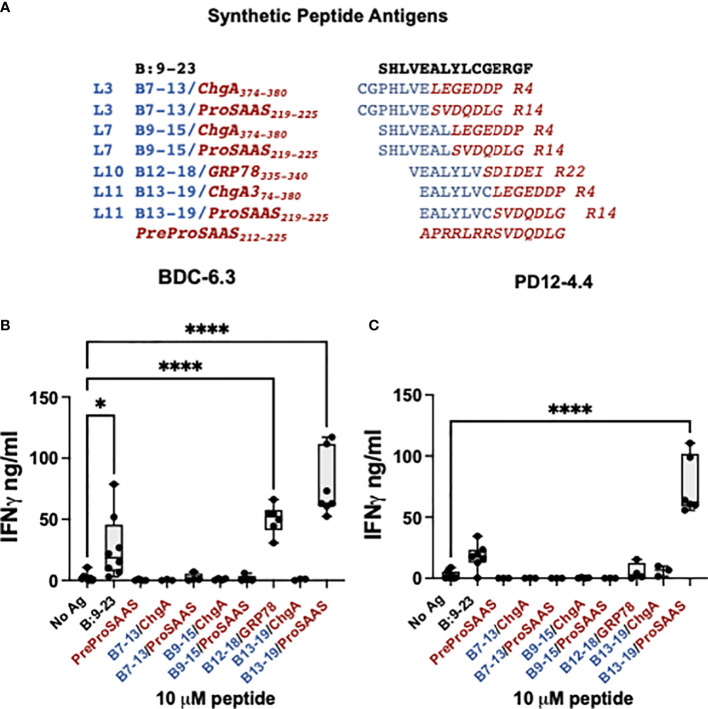
BDC-6.3 IFN-γ response to candidate B-chain HIP antigens. **(A)** Synthetic peptides representing the highest scoring peptides within the Left and Right peptide libraries. Blue = B:9-23 derived peptides, red italics = natural cleavage product peptides from other ß-cell proteins. The PreProSAAS peptide is 14 amino acids spanning a dibasic proteolytic processing site and was included as a negative control. **(B)** IFN-γ responses of T cell clone BDC-6.3 to 10 µM concentrations of B:9-23 or synthetic B-chain HIP candidates. **(C)** IFN-γ responses of T cell clone PD12-4.4 to 10 µM concentrations of B:9-23 or synthetic B-chain HIP candidates. Compiled data is shown for the average of 3-8 experiments (BDC-6.3) or 3-7 experiments (PD12-4.4), with statistical analysis using a one-way ANOVA with Dunnett’s multiple comparison test *P<0.05, ****P<0.0001 where the box defines the interquartile range, and the whiskers indicate minimum and maximum values.

The initial screening of the cross-linked libraries for the insulin-reactive T cell clones BDC-6.3 and PD12-4.4 is shown in **Figures 1B, C**. Individual right peptides (R1-28) crosslinked to a pool of 11 left B chain peptides were used to stimulate IFN-γ production from T cell clones in an antigen assay co-cultured with NOD APCs. The most stimulatory individual right peptide for clone BDC-6.3 was ProSAAS_219-225_ (R14) (**Figure 1B** and **Table S2**). The most stimulatory right peptides for T cell clone PD12-4.4 were ChgA_374-379_ (R5) and ProSAAS_219-225_ (R14)(**Figure 1B** and **Table S2**). We focused on the one positive right peptide pool that was common to both clones, ProSAAS_219-225_ (R14), and the highest scoring individual right peptide pool for PD12-4.4, ChgA_374-379_ (R5). We also observed intermediate IFN-γ responses from both T cell clones to right peptides PC2_616-621_(R20), GRP78_275-280_ (R21) and GRP78_335-340_ (R22). R22 was selected to represent a pool that generated a moderate IFN-γ response. (**Figures 1B, C**, **Table S2**). When Individual left B-chain peptides (L1-11) were crosslinked to two pools of 14 right peptides (R1-14 and R15-28), T cell clone BDC-6.3 gave the highest IFN-γ response to left peptide insB_8-13 _(L3) crosslinked to R15-28. (**Figure 1B**, **Table S2**). Clone PD12-4.4 was most responsive to left peptides insB_10-15 _(L7)_ _and insB_14-19 _(L11) when cross-linked to R15-28 and R1-14 respectively (**Figure 1C**, **Table S2**). Combinations of left and right peptides that stimulated T cell clones BDC-6.3 and PD12-4.4 in the library screen were subsequently synthesized as individual HIPs at high purity for further examination (listed in **Figure 2A**).

### A B-chain HIP is the ligand for the BDC-6.3 T cell clone

To determine the T cell antigen specificity for individual peptides, synthetic peptides 14 amino acids in length and at 95% purity, were obtained from a commercial source and tested for reactivity with the insulin-reactive T cell clones BDC-6.3 and PD12-4.4 (**Figures 2A–C**). The seven B-chain HIP candidates tested were comprised of four B-chain left peptides (B_7-13_, B_9-15_, B_12-18_, and B_13-19_) combined with three right secretory granule peptides (ProSAAS_219-225_, ChgA_374-380_ and GRP78_335-340_) (**Figure 2A**). We measured IFN-γ secretion by T cell clones BDC-6.3 and PD12-4.4 in response to 10 µM concentrations of the synthetic B-chain HIP candidate sequences or the native B:9-23 peptide; data for the BDC-6.3 clone and PD12-4.4 clone is shown in **Figures 2B, C**, respectively. BDC-6.3 responded to two B-chain HIPs, B_13-19_/ProSAAS_219-225_ and InsB/GRP78. PD12-4.4 also responded to B_13-19_/ProSAAS_219-225_ but only weakly to InsB/GRP78 (**Figure 2C**). None of the other synthetic B-chain hybrid peptides elicited a response from either clone despite responses to various pools in the initial screen. Due to the strong response of T cell clone BDC-6.3 to the B_13-19_/ProSAAS_219-225_ HIP (EALYLVC-SVDQDLG), we termed this peptide sequence the 6.3HIP.

**Figure 3:**
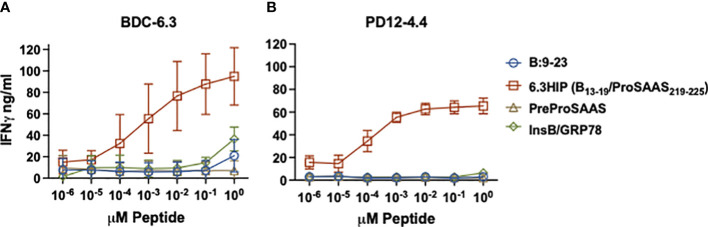
IFN-γ responses of T cell clones to parent peptides and B-chain HIPs. Stimulation of T cell clones BDC-6.3 **(A)** and PD12-4.4 **(B)** with titrations (0.00001 - 10 μM) of individual left (B:9-23) and right (PreProSAAS_212-225_) components of the 6.3HIP (B_13-19_/ProSAAS_219-225_), the 6.3HIP and B-chain HIP InsB/GRP78_335-340_. Blue open circle = B:9-23, red open square = 6.3HIP, brown open triangle = PreProSAAS_212-225_, green open diamond = InsB/GRP78_335-340_. Compiled data is shown for the mean ± SD of 3-5 experiments (BDC-6.3) and 3-4 experiments (PD12-4.4). Data is expressed as IFN-γ ng/ml.

To further characterize the response of BDC-6.3 and PD12-4.4 to the two B-chain HIPs, we assayed IFN-γ responses of these T cell clones to titrations of four peptides: native insulin B:9-23, the 6.3HIP, the B-chain HIP InsB/GRP78 and a peptide spanning the propeptide sequence of ProSAAS (as a negative control). As shown in **Figure 3**, both BDC-6.3 and PD12-4.4 show a strong IFN-γ response to the B-chain 6.3HIP, titrating into the picomolar range. In this assay, the response of T cell clone BDC-6.3 to B-chain HIP InsB/GRP78 was measurable, but only at the 10 µM concentration. Neither of the T cell clones responds to the propeptide ProSAAS_212-225_ sequence, or to the natural cleavage product proSAAS_219-230_ (not shown) even at a concentration of 10 µM.

### Defining the 6.3HIP core epitope

**Figure 4:**
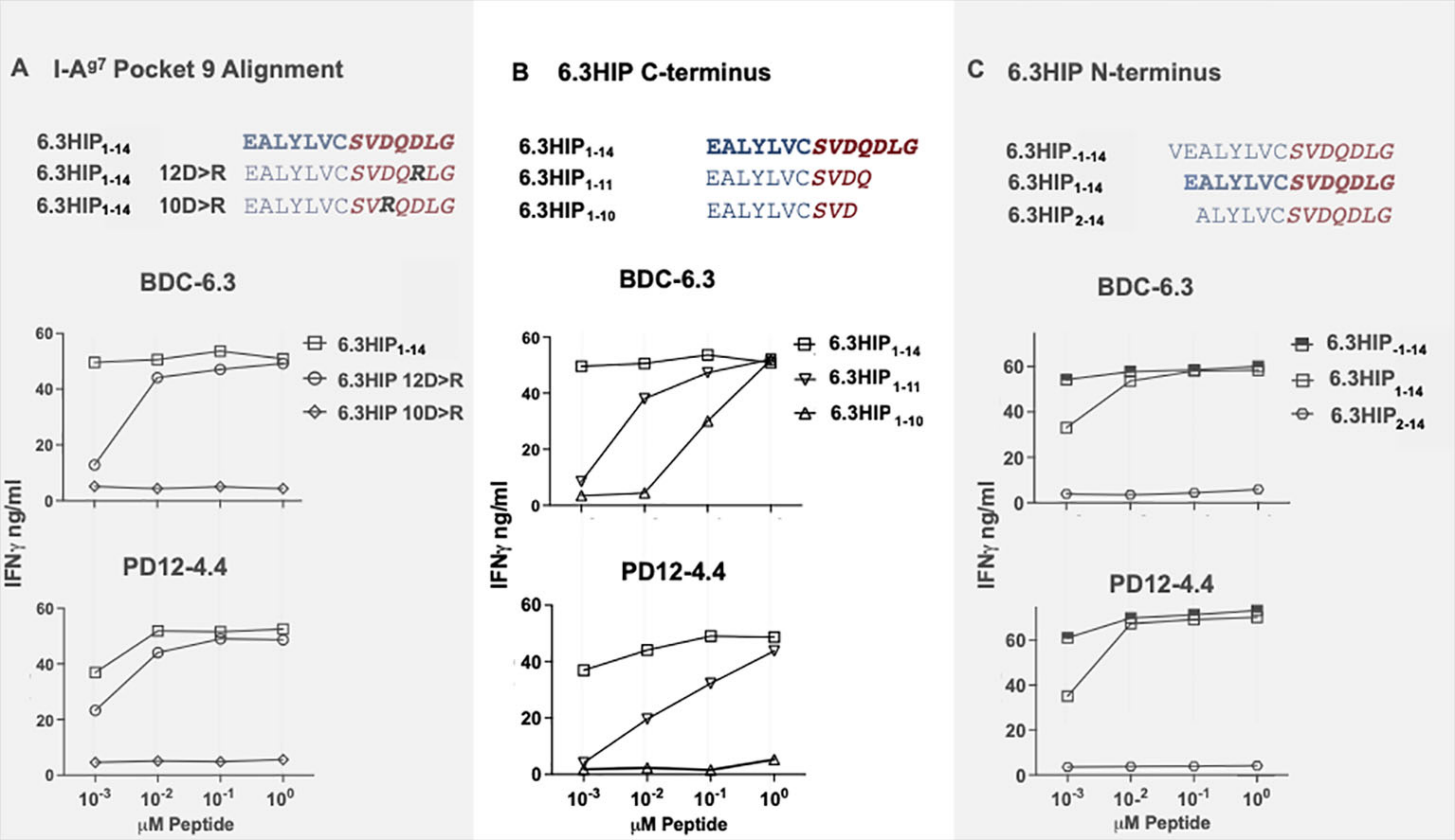
I-A^g7^ binding pocket 9 and optimal limits of the 6.3HIP antigen. **(A)** Determination of 6.3HIP residue occupying p9 of I-A^g7^. IFN-γ ELISA assays of T cell clones BDC-6.3 and PD12-4.4 stimulated with 6.3HIP_1-14_ antigen compared to 6.3HIP substituted with 12D>R or 10D>R. **(B)** Response to C-terminal truncations of the 6.3HIP. IFN-γ production by BDC-6.3 and PD12-4.4 in response antigens 6.3HIP, 6.3HIP_1-11_ and 6.3HIP_1-10_. **(C)** Limits of the 6.3HIP epitope N-terminus. IFN-γ production by BDC-6.3 and PD12-4.4 in response to peptides 6.3HIP. 6.3HIP_-1-14_, and 6.3HIP_2-14_. Blue = B:9-23 sequence, red italics = ProSAAS sequence. Representative data from 4 experiments **(A)**, 6 experiments **(B)**, and 4 experiments **(C)** shown as ng/ml IFN-γ.

The peptide-binding grooves of MHC class II molecules can be described in terms of pockets (p1, p4, p6 and p9) that must accommodate the amino acid side chains of each peptide antigen. It is well-established that NOD mice possess a unique polymorphism at position 57 in the β-chain of the MHC class II molecule I-A^g7^: the aspartic acid (D) present in non-autoimmune BALB/c mice is replaced by serine (S) in NOD mice (49–52). The loss of a negatively charged residue at β57 favors the presentation of epitopes containing a negatively charged amino acid at the p9 position in the peptide binding groove (53), which may explain the unique immunopeptidome presented by I-A^g7^ (54). Since the 6.3HIP contains D in both positions 10 and 12 (**Figure 4A**), we synthesized two mutant 6.3HIP peptides substituting either of the two negatively charged D residues with a positively charged arginine (10D>R or 12D>R) (**Figure 4A**). Our results show that the mutant peptide 6.3HIP 10D>R could not stimulate either the BDC-6.3 or the PD12-4.4 T cell clone, whereas activation of these clones with 6.3HIP 12D>R was comparable to the 6.3HIP (down to 0.1 µM), suggesting that the D at position 10 occupies p9 of I-A^g7^. Furthermore, truncation of 12D at the C-terminus (6.3HIP_1-11_) moderately affected stimulation of both BDC-6.3 and PD12-4.4, suggesting that the 12D residue of the 6.3HIP is not essential for yet enhances antigenicity (**Figure 4B**). Truncation of the glutamine (Q) immediately after 10D (6.3HIP_1-10_) diminished activation of the PD12-4.4 T cell clone, and to a lesser extent BDC-6.3, indicating a critical role of the Q in position 11 (**Figure 4B**). In all cases, differences in reactivity of the T cell clones to altered ligands compared to the 6.3HIP was most apparent at the lowest concentration (.001 µM). We concluded that the minimum contribution from the ProSAAS right peptide is four amino acids, ProSAAS_219-222_.

**Figure 5:**
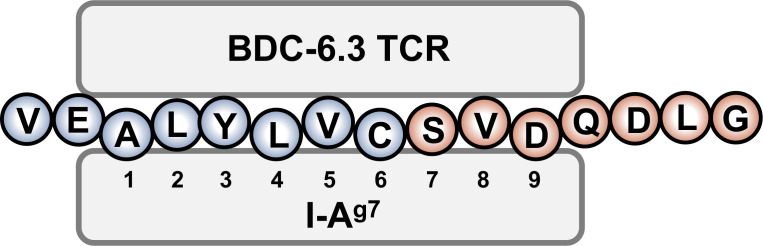
Schematic diagram of the 6.3HIP binding in the trimolecular complex. I-A^g7^ binding pockets 1, 4, and 6 are occupied by insB-chain amino acids 14 (A), 17 (L) and 19 (C), respectively. ProSAAS amino acids 219 (S) and 221 (D) residue in pockets 7 and 9 of I-A^g7^.

The contribution of N-terminal amino acids to the antigenicity of the 6.3HIP was assessed in **Figure 4C**. While truncation of the N-terminal glutamic acid (E) of the 6.3HIP (6.3HIP_2-14_) prevented stimulation of both T cell clones, addition of a valine (V) to the N-terminal sequence of the 6.3HIP potentiated activation of both clones. Even though the E and the V would be predicted to be outside of the binding groove (in position p-1 and p-2, respectively), these data indicate that the N-terminal portion of the 6.3HIP plays an essential role in its activity and define VEALYLCSVDQDLG as the optimal ligand for both T cell clones (**Figure 4C**). The schematic diagram in **Figure 5** illustrates how the 6.3HIP may bind to I-A^g7^ based on our cumulative findings.

### A 6.3HIP- I-A^g7^ tetramer binds insulin-reactive CD4 T cell clones and a sub-population of islet-infiltrating CD4 T cells in NOD mice

**Figure 6:**
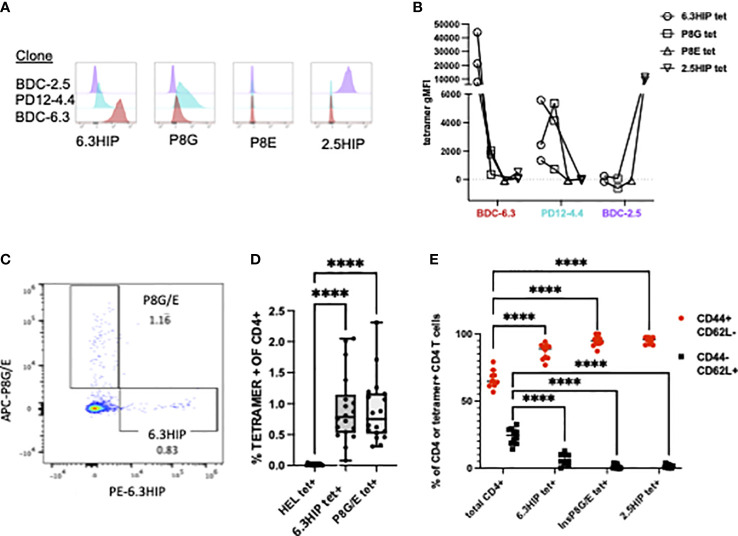
MHC Class II tetramer staining of CD4+ T cell clones and islet infiltrating CD4+ T cells. Representative histograms of **(A)** T cell clones BDC-2.5 (purple), PD12-4.4 (blue), and BDC-6.3 (red) stained with 6.3HIP, P8G, P8E, and 2.5HIP tetramers and the **(B)** geometric MFI from 2-3 experiments with lines connecting tetramer stains conducted on the same day. **(C)** Representative CD4 T cells from NOD islets stained with a mixture of I-A^g7^ tetramers loaded with the P8G/E insulin mimotopes and 6.3HIP, and labeled with APC and PE, respectively. **(D)** Summary of results of the analysis of infiltrating CD4+ T cells from the islet isolations from a total of 18 NOD mice (5 separate islet isolations) stained with hen egg lysozyme (HEL), 6.3HIP and P8G/E tetramers. Data represented as percent of live lin^-^ CD4^+^ T cells positive for each tetramer/animal and analyzed using a one-way ANOVA with Tukey’s multiple comparison test, (****P<0.0001) where the box defines the interquartile range, and the whiskers indicate minimum and maximum values. **(E)** Summary data (n=10) comparing the proportion of CD44+ and CD62L+ in total CD4 T cells or tetramer+ CD4 T cells within islets. A two-way ANOVA was used with Dunnett’s multiple comparison test to assess the antigen experience phenotype of CD4 T cells staining with each tetramer compared to the total CD4 infiltrate as a control (****P<0.0001). Gating strategy for tetramer staining of T cells is provided in **Figure S2**.

MHC class II tetramers can be robust tools for detecting antigen-specific CD4 T cells by flow cytometry. To analyze B-chain HIP tetramer-positive (tet+) T cells in NOD mice, we obtained a tetramer from the NIH Tetramer Core consisting of the 6.3HIP_1-11_ peptide (EALYLVCSVDQ) loaded onto fluorescently labeled I-A^g7^. T cell clones BDC-6.3, PD12-4.4 and BDC-2.5 (as a control), were analyzed for staining with the 6.3HIP tetramer, the two insulin mimotope tetramers (insp8G or insp8E), or the 2.5HIP tetramer. The BDC-6.3 clone stained with the 6.3HIP tetramer with high intensity and only slightly with the insp8G tetramer; no binding of this T cell clone by the insp8E or 2.5HIP tetramer was observed. The T cell clone PD12-4.4 was moderately bound by both the 6.3HIP and p8G tetramers and had no cross reactivity to the insp8E or 2.5HIP tetramers. As expected, BDC-2.5 did not bind either of the B-chain mimotope tetramers or the 6.3HIP tetramer, and only to the cognate 2.5HIP tetramer (**Figures 6A, B**). These data demonstrate that the 6.3HIP tetramer selectively binds the 6.3HIP-reactive T cell clone BDC-6.3 and to a lesser extent PD12-4.4.

It has been established that T cells accumulate in the pancreas only when their cognate antigen is present, suggesting a majority of CD4 T cells in the pancreas have unassigned antigen specificity (15, 55). To determine whether 6.3HIP-reactive T cells were present in the islets of NOD mice, we used the 6.3HIP tetramer to stain cells from dissociated islets of prediabetic NOD mice. As shown in **Figure 6C**, we detected a population of CD4 T cells that stained exclusively with the 6.3HIP tetramer, which represented about 0.75% of the total CD4 infiltrate, a percentage significantly elevated compared to T cells staining with the HEL tet (negative control) and similar to the percentage of the combined insp8G/E tet+ cells (n=18; **Figure 6D**). The islet-infiltrating CD4 cells staining with the 6.3HIP tetramer exhibit an antigen-experienced phenotype (CD44^+^ CD62L^-^) (**Figures 6E**, **S2**), similar to that of 2.5HIP-reactive T cells in islet infiltrates (12). The observation that the 6.3HIP tetramer captures a population of CD4 T cells not stained with either insp8G or insp8E tetramers suggests that this tetramer detects a separate population of disease-relevant insulin-reactive T cells within islets.

## Discussion

Although the insulin B-chain sequence B:9-23 has been widely regarded to be the principal antigenic T cell epitope in the insulin molecule, there is little if any difference in the reactivity of T cells to this epitope when comparing controls and T1D patients (45). Moreover, we observe that insulin-reactive T cell clones typically demonstrate low to moderate responses *in vitro* to the B:9-23 sequence. One possibility could be that the B chain undergoes post-translational modification to become a more potent agonist. We hypothesized that insulin B-chain sequences could fuse with other ß-cell granule peptides through transpeptidation to form B-chain HIPs highly stimulatory to B:9-23-reactive T cells. Combinatorial B-chain HIP libraries were screened with diabetogenic T cell clones responsive to insulin B:9-23 to identify two B-chain HIPs that could stimulate clones BDC-6.3 and PD12-4.4. Our data presented here demonstrate that the 6.3HIP, a B-chain HIP comprised of part of B:9-23 joined to a sequence from ProSAAS, is a potent ligand for BDC-6.3. ProSAAS is an endogenous inhibitor of prohormone convertase 1, required for proteolytic processing of proinsulin to its bioactive form, and serves as a neuroendocrine chaperone (56, 57). Along with insulin, ProSAAS ranks in the top five most abundant proteins within ß-cell secretory granules (58), suggesting that a high concentration and accessibility of these proteins may facilitate formation of the 6.3HIP. We also found that the previously described B:9-23-reactive CD4 T cell clone, PD12-4.4 (9), reacts to the 6.3HIP epitope. To our knowledge this is the first report defining ProSAAS as the source of an islet antigen in autoimmune disease. In addition, the T cell clone BDC-6.3 is slightly activated at high concentrations (10 µM) by a second B-chain HIP, containing a similar fragment of B:9-23 fused to a peptide from GRP78 (which contains two D residues for putative p9 binding), the InsB/GRP78 HIP. Thus, a sequence from B:9-23 may be providing only the N-terminal portion of the epitope recognized by some insulin-reactive T cells. By identifying a hybrid insulin peptide neo-epitope that contains an insulin B:9-23 sequence, these studies may provide an explanation for the apparent epitope dominance of the B chain in autoimmune diabetes, despite the limited ability of the native peptide to bind to I-A^g7^.

We also describe and validate a new tetramer containing the 6.3HIP. This reagent strongly stains the BDC-6.3 T cell clone, and to a lesser degree, the PD12-4.4 T cell clone. In NOD mice, the 6.3HIP tetramer detects a subpopulation of islet-infiltrating CD4 T cells that are distinct from those staining with the insulin tetramers, insp8E and insp8G, which are mimotopes for B:9-23. Our data also indicate that islet-infiltrating CD4 T cells that bound the 6.3HIP tetramer almost exclusively exhibited an antigen-experienced phenotype (CD44^hi^ CD62L^-^) and were found in similar abundance to the combined P8G/P8E tet+ population in the islets of 10-12 wk-old mice. The phenotype of 6.3HIP tet+ cells parallels previous studies showing that nearly all 2.5HIP tet+ T cells found in islets are antigen-experienced in the NOD mouse (12). We speculate that in NOD mice the 6.3HIP tetramer detects a non-redundant population of insulin-reactive CD4 T cells highly specific for a B-chain HIP. However, as illustrated by the tetramer staining of the PD12-4.4 clone, there may exist a population of islet-infiltrating T cells capable of binding to multiple tetramers sharing portions of insulin B:9-23.

Several epitopes within the B:9-23 sequence have been defined for pathogenic, insulin-reactive CD4 T cells in the NOD mouse (9, 11, 32, 34, 59). Unanue and colleagues described subsets of “A” and “B” insulin-reactive CD4 T cells (30). Type “A” T cells recognize the insB:13-21 peptide, generated from the conventional processing and loading of the B chain onto I-A^g7^ in antigen-presenting cells and thymic epithelial cells; these T cells are thought to be deleted by negative selection in the thymus. Type “B” T cells are thought to recognize the B:12-20 peptide after an unconventional exogenous uptake of the truncated peptide and direct loading onto I-A^g7^, an event speculated to only occur in the periphery, affording B:12-20-reactive T cells escape from central tolerance (60, 61). Based on the high relative abundance of B:12-20 tet+ cells in the islets of young mice (6 wk-old), type “B” T cells are thought to be involved in disease initiation (50). T cells specific for the 6.3HIP may also escape central tolerance by only a weak, partial recognition of either the B chain of insulin or ProSAAS in the thymus. Although the T cell clones BDC-6.3 and PD12-4.4 react to insulin B:9-23, they do not respond to the B13-21 nor B12-20 peptides and therefore may represent a distinct subset of insulin-reactive T cells, responding to hybrid insulin peptides.

The specificity of I-A^g7^-restricted T cell clones reactive to insB:9-23 is thought to be influenced by both common utilization of certain TCRα variable segments as well as the composition of TCRβ complementarity-determining region 3 (CDR3)(**Tables 1**, **S1**) (62). The TCRα variable segments TRAV5D-4 and TRAJ53, used in both PD12-4.4 and BDC-6.3, are preferentially enriched in B:9-23-reactive T cells (63–65). While the PD12-4.4 and BDC-6.3 clones also share the same TRBV5 segment, they differ in N-region additions within the CDR3β and BDC-6.3 contains a negatively charged E residue, in the +3 position of the CDR3β, shown to be highly enriched in insB:12-20-specific T cells (50, 66). Although the TCRα and β chains of BDC-6.3 and PD12-4.4 have sequences in common to other B:9-23-reactive T cells, our tetramer and mutagenesis studies indicate that these insulin-reactive T cell clones react strongly to a B-chain HIP.

Our data show that 6.3HIP-reactive T cells are present within the islet and are antigen-experienced. However, it may be that the balance between regulatory and effector phenotype of T cells is a stronger determinant of their role in disease. We previously showed that there is a marked difference in phenotype between the highly inflammatory 2.5HIP tet+ T cells and insp8G tet+ T cells which were enriched in regulatory T cells (Tregs) (12). A similar result was obtained in a comparison of islet-infiltrating 6.9HIP tet+ versus insB:12-20 tet+ cells by single-cell RNA sequencing, wherein a subset of insulin B12-20 tet+ cells, but not 6.9HIP tet^+^ cells, were found to exhibit a Treg expression signature (13). It remains to be established whether in the islet infiltrate any 6.3HIP tet+ or other B-chain HIP-reactive T cells exhibit a Treg phenotype or are effector T cells only, like 2.5HIP and 6.9HIP tet+ cells.

## Data availability statement

The original contributions presented in the study are publicly available. This data can be found here: https://doi.org/10.6084/m9.figshare.20092523.v1

## Ethics statement

The animal study was reviewed and approved by University of Colorado Institutional Animal Care and Use Committee.

## Author contributions

JW and JD contributed to design of the study, conducted experiments, analyzed the data, and wrote the manuscript. GB and AH maintained T cell clone cultures and conducted antigen assays. MD generated the B-chain HIP cross-linked libraries. MN generated and interpreted TCR sequencing data. TD was a consultant for and provided the B-chain HIP libraries. RB contributed to design of the study, data analysis and writing of the manuscript. KH oversaw design of the study, critically reviewed and edited the manuscript. All authors contributed to the article and approved the submitted version.

## Funding

This project is supported by grants from the NIH (R01DK081166, R01DK122566, R21AI133059, and 5T32DK120520) and the University of Colorado Diabetes Research Center award P30DK116073.

## Acknowledgments

All tetramers used in this study were obtained from the NIH Tetramer Core. The authors are grateful for critical discussion of the project with members of the Haskins and Delong Labs. We thank MN for TCR sequencing. We appreciate the help of Scott Beard in preparing islets and Kaitlin Reyes in the maintenance of the mouse colony.

## Conflict of interest

The authors declare that the research was conducted in the absence of any commercial or financial relationships that could be construed as a potential conflict of interest.

## Publisher’s note

All claims expressed in this article are solely those of the authors and do not necessarily represent those of their affiliated organizations, or those of the publisher, the editors and the reviewers. Any product that may be evaluated in this article, or claim that may be made by its manufacturer, is not guaranteed or endorsed by the publisher.
