# Supplementary material for: Insulin B-chain hybrid peptides are agonists for T cells reactive to insulin B:9-23 in autoimmune diabetes
Source: Front Immunol. 2022 Aug 10;13:926650. doi: 10.3389/fimmu.2022.926650 (PMC9399855; doi:10.3389/fimmu.2022.926650)
Supplement: Supplementary file 1 [file DataSheet_1.zip › Data Sheet 1 (17)/Data Sheet 1/FigS1.pdf]

	Left peptides	
B1	Ins1B6-11	Ac-RRRGLCGPHL
B1	Ins1B6-11	Ac-RRRGCGPHLV
B3	Ins1B8-B13	Ac-RRRGGPHLVE
B4	Ins2B6-B11	Ac-RRRGLCGSHL
B5	Ins2B7-B12	Ac-RRRGCGSHLV
B6	Ins2B8-B13	Ac-RRRGGSHLVE
B7	Ins1/2B10-B15	Ac-RRRGHLVEAL
B8	Ins1/2B11-B16	Ac-RRRGLVEALY
B9	InsB1/B12-B17	Ac-RRRGVEALYL
B10	Ins1/2B13-B18	Ac-RRRGEALYLY
B11	Ins1/2B14-B19	Ac-RRRGALYLVC

	Right peptides	
R1	ChgC 219-224	ERVDEE
R2	ChgC 300-305	ESKDQL
R3	ChgA 233-238	AKQEEK
R4	ChgA 358-363	WSRMDQ
R5	ChgA 374-379	LEGEDD
R6	ChgA 435-440	AEDQEL
R7	ChgB 21-26	APVDNR
R8	ChgB 64-69	SGKEVK
R9	ChgB 186-191	HIEDSG
R10	ChgB 386-391	NHPDSE
R11	ChgB 438-443	LLDEGH
R12	ChgB 538-543	GNPDDS
R13	ChgB 575-579	PFSEDV
R14	ProSAAS 219 -224	SVDQDL
R15	IAPP 78-83	NAARDP
R16	PC2 59-64	LPFAEG
R17	PC2 109-114	GYRDIN
R18	PC2 419-424	NQLHDE
R19	PC2 539-544	PRDDDS
R20	PC2 616-621	QELEEE
R21	GRP78 275-280	TGKDVR
R22	GRP78 335-340	SDIDEI
R23	GRP78 623-628	ELEEIV
R24	7B2 167-172	LLYEKM
R25	Scg3 23-28	FPKPEG
R26	Scg3 119-124	LIDEYD
R27	Scg3 429-434	GNKEDY
R28	C-peptide 1-11	huEAEDLQVGARR

Supplementary Figure 1. B-chain and secretory granule peptides used in HIP cross-linked libraries. Left peptides B1-11 were acquired with acetyl-blocked N-termini, three R residues for enhanced solubility and a glycine for a spacer before the 6 amino acid B-chain sequence shown. Right peptides are 6 amino acid peptides from the secretory granule or endoplasmic reticulum that are the N-termini of putative dibasic cleavage products.
