# Supplementary material for: Insulin B-chain hybrid peptides are agonists for T cells reactive to insulin B:9-23 in autoimmune diabetes
Source: Front Immunol. 2022 Aug 10;13:926650. doi: 10.3389/fimmu.2022.926650 (PMC9399855; doi:10.3389/fimmu.2022.926650)
Supplement: Supplementary file 1 [file DataSheet_1.zip › Data Sheet 1 (17)/Data Sheet 1/FigS2.pdf]

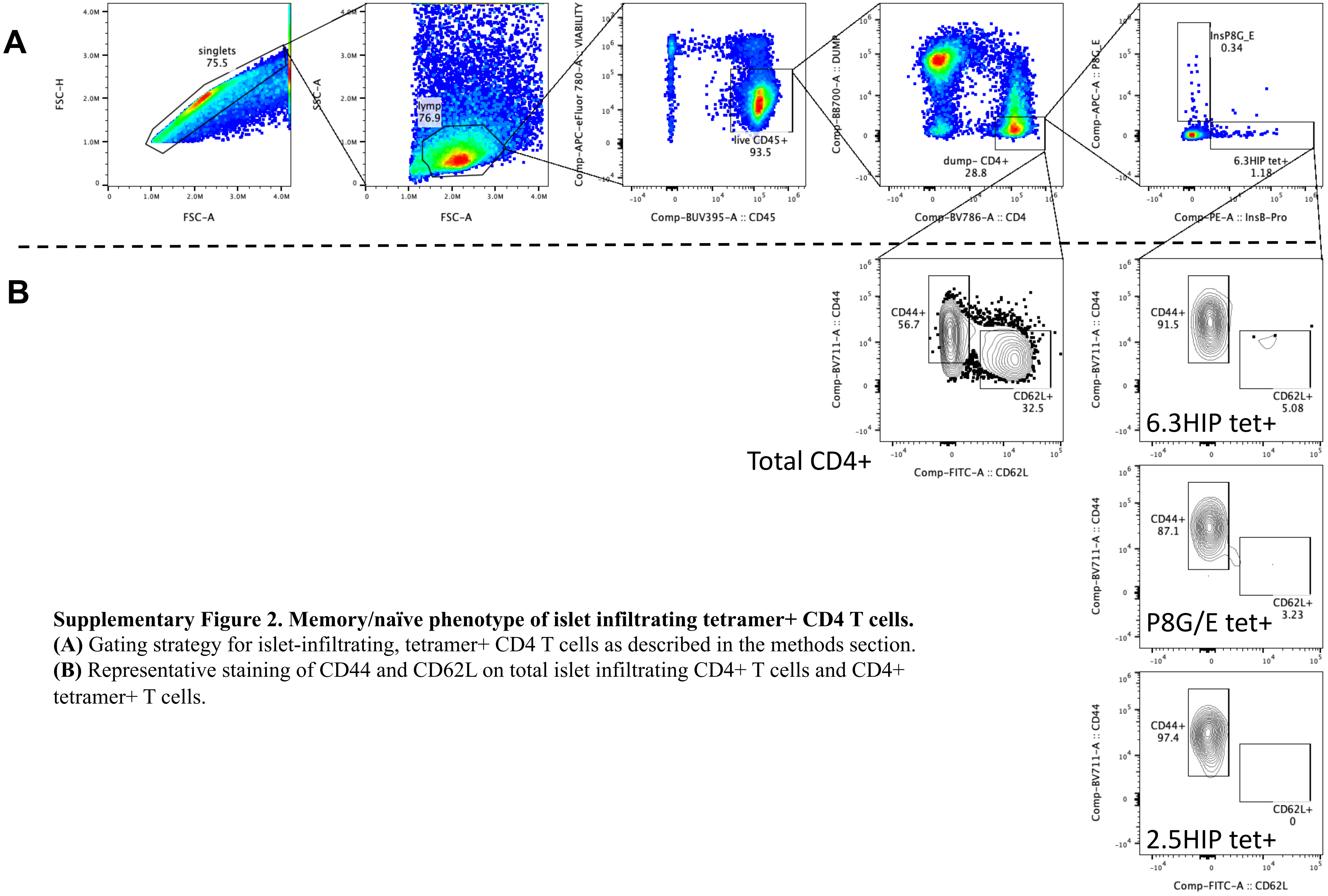

Supplementary Figure 2. Memory/naïve phenotype of islet infiltrating tetramer+ CD4 T cells. **(A)** Gating strategy for islet-infiltrating, tetramer+ CD4 T cells as described in the methods section. **(B)** Representative staining of CD44 and CD62L on total islet infiltrating CD4+ T cells and CD4+ tetramer+ T cells.
