# Supplementary material for: Insulin B-chain hybrid peptides are agonists for T cells reactive to insulin B:9-23 in autoimmune diabetes
Source: Front Immunol. 2022 Aug 10;13:926650. doi: 10.3389/fimmu.2022.926650 (PMC9399855; doi:10.3389/fimmu.2022.926650)
Supplement: Supplementary file 1 [file DataSheet_1.zip › Data Sheet 1 (17)/Data Sheet 1/Table S2.pdf]

BDC-6.3

Right Pool	Ins1B$_{6-11}$	Ins1B$_{7-12}$	Ins1B$_{8-13}$	Ins2B$_{6-11}$	Ins2B$_{7-12}$	Ins2B$_{8-13}$	B$_{10-15}$	B$_{11-16}$	B$_{12-17}$	B$_{13-18}$	B$_{14-19}$	B-chain Peptide			no antigen
R1-14	0.185	0.154	0.172	0.154	0.159	0.162	0.151	0.15	0.171	0.163	0.146				0.137
R15-28	0.159	0.164	0.189	0.155	0.168	0.177	0.162	0.162	0.157	0.175	0.165				no antigen
															0.181
Left Pool	ChgC$_{219-224}$	ChgC$_{300-305}$	ChgA$_{233-238}$	ChgA$_{358-363}$	ChgA$_{374-379}$	ChgA$_{435-440}$	ChgB$_{21-26}$	ChgB$_{64-69}$	ChgB$_{186-191}$	ChgB$_{386-391}$	ChgB$_{438-443}$	ChgB$_{538-543}$	ChgB$_{575-579}$	ProSAAS$_{219-224}$	R1-14
L1-11	0.197	0.213	0.205	0.221	0.235	0.259	0.231	0.216	0.199	0.199	0.194	0.218	0.213	0.28	
L1-11	IAPP$_{78-83}$	PC2$_{59-64}$	PC2$_{109-114}$	PC2$_{419-424}$	PC2$_{539-544}$	PC2$_{616-621}$	GRP78$_{275-280}$	GRP78$_{335-340}$	GRP78$_{623-628}$	7B2$_{167-17}$2	Scg3$_{23-28}$	Scg3$_{119-124}$	Scg3$_{429-434}$	huC-peptide$_{1-11}$	R15-28
	0.196	0.202	0.196	0.211	0.196	0.229	0.229	0.221	0.174	0.195	0.245	0.218	0.196	0.203	

PD12-4.4

Right Pool	Ins1B$_{6-11}$	Ins1B$_{7-12}$	Ins1B$_{8-13}$	Ins2B$_{6-11}$	Ins2B$_{7-12}$	Ins2B$_{8-13}$	B$_{10-15}$	B$_{11-16}$	B$_{12-17}$	B$_{13-18}$	B$_{14-19}$	B-chain Peptide			no antigen
R1-14	0.139	0.143	0.162	0.144	0.151	0.15	0.156	0.138	0.144	0.153	0.169				0.114
R15-28	0.156	0.166	0.157	0.134	0.136	0.161	0.168	0.148	0.153	0.139	0.131				no antigen
															0.148
Left Pool	ChgC$_{219-224}$	ChgC$_{300-305}$	ChgA$_{233-238}$	ChgA$_{358-363}$	ChgA$_{374-379}$	ChgA$_{435-440}$	ChgB$_{21-26}$	ChgB$_{64-69}$	ChgB$_{186-191}$	ChgB$_{386-391}$	ChgB$_{438-443}$	ChgB$_{538-543}$	ChgB$_{575-579}$	ProSAAS$_{219-224}$	R1-14
L1-11	0.137	0.154	0.139	0.133	0.191	0.155	0.157	0.141	0.133	0.145	0.128	0.127	0.125	0.169	
L1-11	IAPP$_{78-83}$	PC2$_{59-64}$	PC2$_{109-114}$	PC2$_{419-424}$	PC2$_{539-544}$	PC2$_{616-621}$	GRP78$_{275-280}$	GRP78$_{335-340}$	GRP78$_{623-628}$	7B2$_{167-17}$2	Scg3$_{23-28}$	Scg3$_{119-124}$	Scg3$_{429-434}$	huC-peptide$_{1-11}$	R15-28
	0.147	0.128	0.135	0.131	0.124	0.139	0.137	0.139	0.115	0.126	0.119	0.119	0.121	0.124	

Table S2. Absorbance Values for IFN-γ ELISA for T cell clones BDC-6.3 and PD12-4.4 using B-chain Left and Right HIP libraries as antigen. Left B-chain peptides are shown in blue, Right β-cell peptides shown in red. Background absorbance measured as ”no antigen”. Absorbance was measured @ 415 nm.
