# Supplementary material for: Insulin B-chain hybrid peptides are agonists for T cells reactive to insulin B:9-23 in autoimmune diabetes
Source: Front Immunol. 2022 Aug 10;13:926650. doi: 10.3389/fimmu.2022.926650 (PMC9399855; doi:10.3389/fimmu.2022.926650)
Supplement: Supplementary file 1 [file DataSheet_1.zip › Data Sheet 1 (17)/Data Sheet 1/TableS1.pdf]

T cell Clone	TCR Chain	V-gene, allele	J-gene, allele	Amino acid junction	D-gene, allele	Junction sequence	Sequence
BDC-6.3	BDC 6.3 **α**	TRAV5D-4/5N-4	TRAJ53	CAASAVGSGGSNYKLTF		tgtgctgcaagtgcagttgggagtggaggcagcaattacaaactgacattt	cacccagcctggagactcagccatgtacttctgtgctgcaagtgcagttgggagtggaggcagcaattacaaactgacatttgggaaaggaactctcttaactgtgactccaaacatccagaacccagaacctgctgtgtacgtctgat
	BDC 6.3 β	TRBV5	TRBJ2-7	CASSQEGGGNEQYF	TRBD2	tgtgccagcagccaagagggggggggcaatgaacagtacttc	gccgtggatccagaagactcagctgtctatttttgtgccagcagccaagagggggggggcaatgaacagtacttcggtcccggcaccaggctcacggttttagaggatctgagaaatgtgactccacccaaggtctccttgtgtctgat
PD 12.4.4	PD 12.4.4 **α**	TRAV5D-4/5N-4	TRAJ53	CAASASGGSNYKLTF		tgtgctgcaagtgcaagtggaggcagcaattacaaactgacattt	cacagacacccagcctggagactcagccatgtacttctgtgctgcaagtgcaagtggaggcagcaattacaaactgacatttgggaaaggaactctcttaactgtgactccaaacatccagaacccagaacctgctgtgtaccgagcgt
	PD 12.4.4 β	TRBV5	TRBJ2-2	CASSQDTNTGQLYF	TRBD1	tgtgccagcagccaagacacaaacaccgggcagctctacttt	gccgtggatccagaagactcagctgtctatttttgtgccagcagccaagacacaaacaccgggcagctctactttggtgaaggctcaaagctgacagtgctggaggatctgagaaatgtgactccacccaaggtctccttgtcgagcgt

Table S1. DNA sequence of T cell receptor (TCR) of T cell clones BDC-6.3 and PD12-4.4. V, J, and D genes and alleles are listed for T cell clones BDC-6.3 and PD12-4.4. The amino acid junction sequences, DNA junction sequences, and TCR **α** and β chain DNA sequences are listed for each clone.
