# Supplementary figures and images for: Insulin B-chain hybrid peptides are agonists for T cells reactive to insulin B:9-23 in autoimmune diabetes
Source: Front Immunol. 2022 Aug 10;13:926650. doi: 10.3389/fimmu.2022.926650 (PMC9399855; doi:10.3389/fimmu.2022.926650)

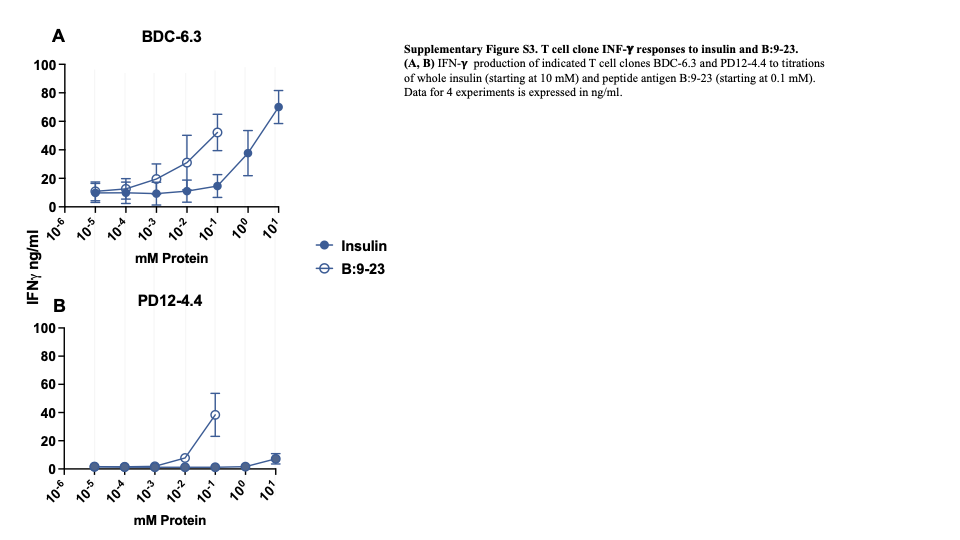

Supplement: Supplementary file 1 [file DataSheet_1.zip › Data Sheet 1 (17)/Data Sheet 1/FigS3.tiff]
